# Supplementary material for: Splice-Junction-Based Mapping of Alternative Isoforms in the Human Proteome
Source: Cell Rep. Author manuscript; Available in PMC 2020 Jan 15. (PMC6961840; doi:10.1016/j.celrep.2019.11.026)
Supplement: 2 [file NIHMS1546469-supplement-2.zip › DF1/PXD006675/VentricularSeptum/VentricularSeptum_37_TPM3_AISEELDHALNDMTSGS.pdf]

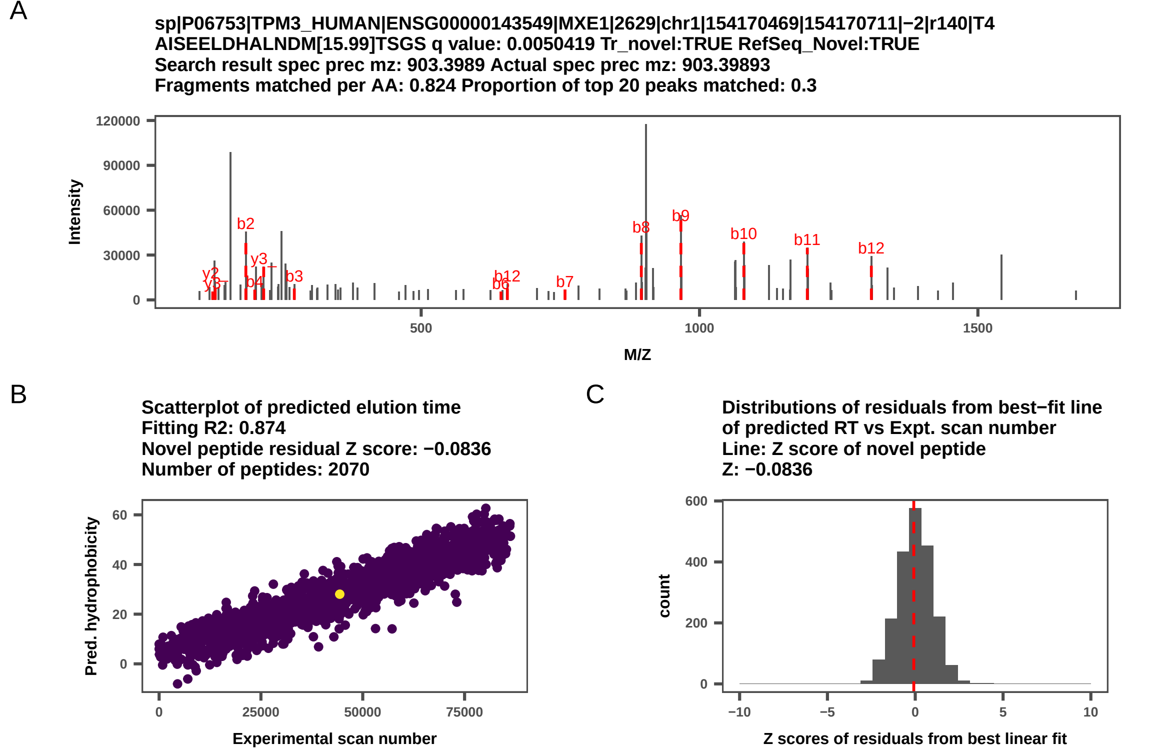
A
sp|P06753|TPM3_HUMAN|ENSG00000143549|MXE1|2629|chr1|154170469|154170711|−2|r140|T4
AISEELDHALNDM[15.99]TSGS q value: 0.0050419 Tr_novel:TRUE RefSeq_Novel:TRUE
Search result spec prec mz: 903.3989 Actual spec prec mz: 903.39893
Fragments matched per AA: 0.824 Proportion of top 20 peaks matched: 0.3
Intensity
120000
90000
60000
30000
0
b2
y2
y3
y3
b4
b3
b12
b6
b7
b8
b9
b10
b11
b12
500
1000
1500
M/Z
B
Scatterplot of predicted elution time
Fitting R2: 0.874
Novel peptide residual Z score: −0.0836
Number of peptides: 2070
Pred. hydrophobicity
60
40
20
0
0
25000
50000
75000
Experimental scan number
C
Distributions of residuals from best−fit line
of predicted RT vs Expt. scan number
Line: Z score of novel peptide
Z: −0.0836
count
600
400
200
0
−10
−5
0
5
10
Z scores of residuals from best linear fit
